# miR-383-3p and miR-6951-3p activate cell proliferation through the regulation of genes related to hypertelorism

**DOI:** 10.3389/fcell.2025.1587052

**Published:** 2025-07-24

**Authors:** Chihiro Iwaya, Junichi Iwata

**Affiliations:** ^1^Department of Orthodontics and Pediatric Dentistry, School of Dentistry, University of Michigan, Ann Arbor, MI, United States; ^2^Department of Diagnostic & Biomedical Sciences, School of Dentistry, The University of Texas Health Science Center at Houston, Houston, TX, United States

**Keywords:** craniofacial development, MicroRNAs, hypertelorism, birth defects, cranial neural crest cells

## Abstract

Hypertelorism, characterized by an abnormal increase in the distance between the eyes, is often associated with various congenital birth defects. While there is increasing evidence suggesting common underlying mechanisms for hypertelorism, the role of microRNAs (miRNAs)—short noncoding RNAs that suppress target genes by inhibiting translation and degrading mRNA—in the condition’s pathogenesis remains unclear. This study aimed to identify the miRNAs associated with hypertelorism in mice. By searching the Mouse Genome Informatics (MGI) database and reviewing full-text references, we identified a total of 31 genes potentially related to hypertelorism. Advanced bioinformatics analyses revealed nine miRNAs that may regulate these genes. We experimentally evaluated candidate miRNAs in assays of cell proliferation and target gene regulation in primary cells isolated from developing frontonasal process mouse embryonic frontonasal mesenchymal and O9-1 cells, a murine neural crest cell line. Our findings indicated that overexpression of either miR-383-3p or miR-6951-3p stimulated cell proliferation, whereas miR-7116-3p and miR-124-3p did not have this effect. Additionally, we confirmed that miR-383-3p and miR-6951-3p regulated the expression of a set of hypertelorism-related genes in a dose-dependent manner. These results suggest that miR-383-3p and miR-6951-3p play significant roles in the development of hypertelorism.

## 1 Introduction

The frontonasal prominence (FNP) forms the midfacial region, including the forehead, nose, philtrum of the upper lip, and primary palate. It starts to develop at 4 weeks of gestation in humans and embryonic day (E) 9.5 in mice and the FNP forms a pair of medial and lateral nasal prominences (MNP and LNP), which develop into the philtrum, primary palate and nasal pits, and fuses with the maxillary prominence by 6 weeks of gestation in humans and E12.5 in mice (Som and Naidich, 2013; Suzuki et al., 2016). Various signaling pathways, including sonic hedgehog (SHH) and fibroblast growth factor (FGF), play crucial roles in FNP outgrowth through tissue-tissue interactions (Suzuki et al., 2016; Cobourne et al., 2009).

In humans, facial morphological variants are evaluated in a dysmorphology exam with precise anthropometric facial measurements and cephalometric analyses (Myers et al., 2020). Hypertelorism (a.k.a. orbital hypertelorism) is characterized by an abnormally increased distance between the orbits, yielding more than the 95th percentile on normative anthropometric values in inner canthal distance, outer canthal distance, and interpupillary distance (Cohen et al., 1995). Hypertelorism is often associated with other congenital birth defects such as midline facial cleft, craniosynostosis syndromes (e.g., Apert syndrome), and encephaloceles (Kimonis et al., 2007). Morphometric analyses show a strong association between facial morphology and/or variations in genetic architecture in humans (Sero et al., 2019; Claes et al., 2018; Knol et al., 2022; Xiong et al., 2019; White et al., 2021). For instance, interorbital distance is significantly associated with human genomic variants at a single base position as single nucleotide polymorphisms (SNPs) at *RPS12*, *EYA4* (6p21.2; rs5880172) (Claes et al., 2018), and *STX18-AS1* (4p16.2; rs13117653) (White et al., 2021). Recent studies show that microRNAs (miRNAs), non-coding short single RNAs with 18–25 nucleotides, are involved in the cause of various birth defects through spatiotemporal gene regulation (Iwaya et al., 2023). miRNAs inhibit the expression of multiple target genes (Huang et al., 2020) and play a role in craniofacial development in humans and mice (Iwaya et al., 2023). FNP development is affected by the expansion of the forebrain as well as cell proliferation, apoptosis, and migration. Hypertelorism is caused by any disruption in an orbital movement towards the midline and hypergrowth of the region between eyes during frontonasal development. This study aims to identify specific miRNAs potentially associated with hypertelorism in mice. In this study, we focus on increased cell proliferation related to overgrowth of the region between eyes, one of the potential mechanisms of hypertelorism. This step could significantly advance our understanding of this condition and potentially lead to new treatment strategies.

## 2 Materials and methods

### 2.1 Mouse genome informatics database search

The Mouse Genome Informatics (MGI) database (https://www.informatics.jax.org) was used to search genes related to hypertelorism in mice, with search terms ‘hypertelorism’ and ‘wide distance between eyes.’ The genes from the MGI search were further evaluated through the references cited.

### 2.2 Bioinformatic analysis

miRTarbase, miRbase, MirDB, and TargetScan were used to predict miRNA-target gene regulation, as previously described (Suzuki et al., 2018a). The significance level of the shared genes between miRNA-target genes and genes related to hypertelorism was examined with Fisher’s exact test (Riol et al., 2020). The Bonferroni collection was used for multiple test correction (Benjamini et al., 2001). MGI clustering analysis, the Kyoto Encyclopedia of Genes and Genomes (KEGG) pathway analysis, and the Gene Ontology (GO) enrichment analysis were conducted using ShinyGO 0.77 (http://bioinformatics.sdstate.edu/go/) for biological process (BP), cell component (CC), and molecular function (MF) (Ge et al., 2020; Luo and Brouwer, 2013) for applying the biological pathway. Significantly enriched categories for the genes were filtered with a false discovery rate (FDR)-adjusted *p*-value <0.05 in a hypergeometric test and at least four genes related to hypertelorism. Excessively general GO terms below hierarchical level 4 were excluded.

### 2.3 Cell culture

C57BL/6J mice were ostained from The Jackson Laboratory. Primary mouse embryonic frontonasal mesenchymal (MEFM) cells were isolated from the frontonasal process of C57BL/6J embryos on E10.5. The frontonasal process was dissected in sterile Dulbecco’s Phosphate-Buffered Saline (D-PBS) and then suspended into single-cell suspensions using 0.25% trypsin and 0.05% EDTA for 10 min at 37°C in an atmosphere containing 5% CO_2_. The animal protocol (PRO00011979) was approved by the Animal Welfare Committee (AWC) and the Institutional Animal Care and Use Committee (IACUC) of the University of Michigan. MEFM cells were maintained in Dulbecco’s Modified Eagle’s Medium (DMEM) with high glucose (Sigma-Aldrich) supplemented with 10% fetal bovine serum, penicillin and streptomycin, β-mercaptoethanol, MEM nonessential amino acids, L-glutamine, and sodium pyruvate at 37°C in a humidified atmosphere with 5% CO_2_. O9-1 cells, a murine neural crest cell line (SCC049, Millipore Sigma), were maintained under embryonic stem cell medium (ES-101-B, Millipore Sigma) at 37°C in a humidified atmosphere with 5% CO_2_, as previously described (Yan et al., 2022).

### 2.4 Cell proliferation assay

MEFM or O9-1 cells were treated with a mimic for a negative control (4464061; mirVana miRNA mimic, Thermo Fisher Scientific), miR-383-3p, miR-6951-3p, miR-7116-3p, or miR-124-3p (4464066; mirVana miRNA mimic), or an inhibitor for a negative control (4464079; mirVana miRNA mimic), miR-383-3p, miR-6951-3p, miR-7116-3p or miR-124-3p (4464084; mirVana miRNA inhibitor), using the Lipofectamine RNAiMAX transfection reagent (Thermo Fisher Scientific) according to the manufacturer’s protocol. Cell proliferation was measured using the Cell Counting Kit 8 (Dojindo Molecular Technologies, Inc.) at 24, 48, and 72 h after each treatment (*n* = 6 per group), as previously described (Yan et al., 2022).

### 2.5 Immunocytochemistry

MEFM or O9-1 cells were seeded in an Ibidi 4-well slide (80426; Ibidi) at a density of 1 × 10^5^ cells per well for MEFM cells and 5 × 10^4^ cells per well for O9-1 cells, using 1 mL medium per well. Once the cell density reached 70%, the cells were treated with a mimic for negative control (4464061; mirVana miRNA mimic, Thermo Fisher Scientific), miR-28a-5p, miR-302a-3p, miR-302b-3p, or miR-302d-3p (4464066; mirVana miRNA mimic). Bromodeoxyuridine (BrdU) incorporation assays were conducted, as previously described (Suzuki et al., 2018b). A total of ten randomly selected fields from three independent experiments was used for the quantification of BrdU-positive cells. Immunocytochemical analysis for Ki-67 was performed using mouse monoclonal Ki-67 antibody (ab16667, Abcam, 1:600), as previously described (Suzuki et al., 2018b). Color images were obtained using a light microscope (BX43, Olympus) (*n* = 6 per group). Terminal 2′-deoxyuridine, 5′-triphosphate (dUTP) nick-end labeling (TUNEL) staining was performed using the Click-iT Plus TUNEL Assay (C10618, Molecular Probes), as previously described (Suzuki et al., 2018c). TUNEL-positive cells were quantified using five randomly selected fields from two independent experiments (total 10 fields). Fluorescent images were captured using a confocal microscope (Ti-C2, Nikon), while color images were obtained using a light microscope (BX43, Olympus).

### 2.6 Quantitative RT-PCR

MEFM or O9-1 cells were treated with either mimic or inhibitor for miR-383-3p, miR-6951-3p, miR-7116-3p, miR-124-3p, or a negative control, and gene expression was analyzed by quantitative RT-PCR (qRT-PCR), as previously described (*n* = 6 each group) (Yan et al., 2022). The PCR primers used in this study are listed in Supplementary Table S1.

### 2.7 TaqMan assay

Pregnant C57BL/6J mice were euthanized on E10.5, E11.5, and E12.5. The frontonasal processes and other relevant tissues were dissected from the embryos. These tissues were processed for total RNA extraction using the miRNeasey mini kit (217004, Qiagen) and converted to cDNA using the TaqMan microRNA reverse transcription kit (4366596, Thermo Fisher Scientific). miRNA expression levels were measured using the TaqMan system (Thermo Fisher Scientific) with probes for miR-124-3p (mmu480901_mir), miR-383-3p (mmu481150_mir), miR-6951-3p (mmu482850_mir), miR-7116-3p (mmu482410_mir), and U6 (001973), following the manufacturer’s instructions. A total of six samples from different litters were analyzed.

### 2.8 Statistical analysis

One-way analysis of variance (ANOVA) with the Tukey–Kramer *post hoc* test was used for multiple comparisons and two-way ANOVA was used for cell proliferation assays. Cell proliferation assays were analyzed using a two-way ANOVA. All experimental data were analyzed using the Prism software version 10 (GraphPad Software) and *p*-value <0.05 was considered statistically significant.

## 3 Results

### 3.1 Identification of a set of genes related to hypertelorism in mice

**FIGURE 1:**
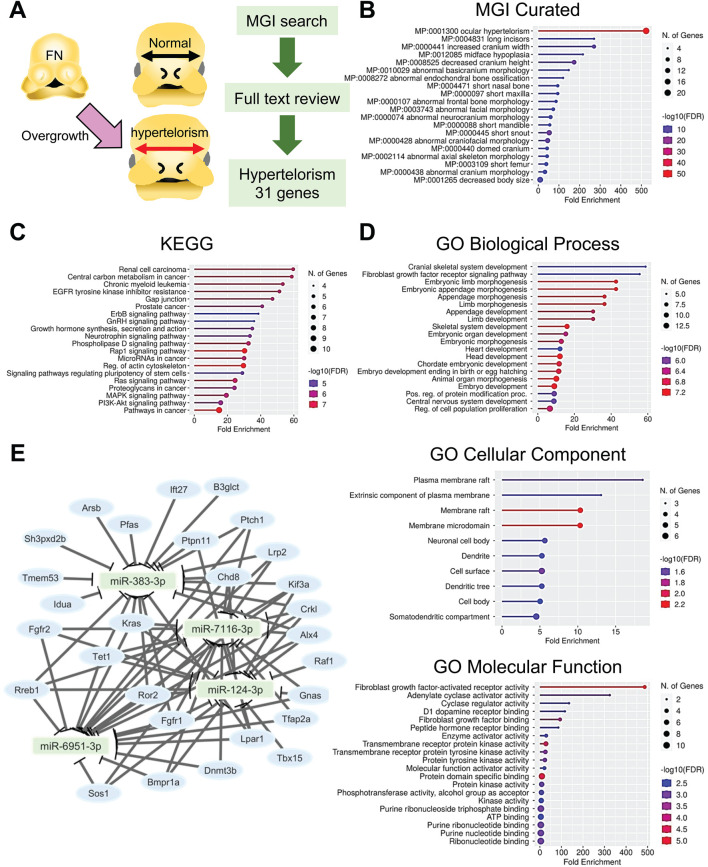
Prediction of hypertelorism-related miRNAs. **(A)** Drawing picture and flowchart for the identification of genes related to hypertelorism. **(B)** Lollipop graphs for MGI gene ontology curation for the genes related to hypertelorism. **(C)** Lollipop graphs for KEGG analysis for the genes related to hypertelorism. **(D)** Lollipop graphs for GO analysis. BP, biological process; CC, cellular component; and MF, molecular function. Circle size indicates the number of genes involved. Color code represents the -log10 FDR; low (blue) to high (red). **(E)** miRNA-gene network analysis. Square (light green) represents the predicted miRNAs. Light blue circle represents genes related to hypertelorism.

The information on genes related to hypertelorism in mice was collected through an MGI database search with reference literature validation. A total of 31 genes were confirmed, following full-text review, to be related to hypertelorism in mouse genetic models (Figure 1A; Table 1). The genes related to hypertelorism included either genes specific to hypertelorism (nonsyndromic hypertelorism genes) or those associated with broad craniofacial malformations (syndromic hypertelorism genes), caused by not only increased cell proliferation in the frontonasal region but also other craniofacial and/or brain anomalies. Genes were most significantly enriched in ocular hypertelorism (MP:0001300, enrichment FDR; 3.57 × 10^−52^) in the MGI clustering (Figure 1B; Supplementary Table S2). These genes were further grouped, according to function and pathway, with the KEGG pathway analysis; the genes were enriched in cancers, central carbon metabolism, EGFR tyrosine kinase inhibitor resistance, Gap junction, endocrine resistance, growth hormone synthesis, secretion and action, signaling pathways regulating pluripotency of stem cells, small GTPase RAP1 signaling pathway, phospholipase D signaling pathway, proteoglycans, miRNAs, regulation of actin cytoskeleton, RAS GTPase signaling pathway, mitogen-activated protein kinase (MAPK) signaling pathway, and phosphoinositide 3-kinase (PI3K)-AKT signaling pathway (Figure 1C; Supplementary Table S3). Importantly, the KEGG pathway analysis highlighted miRNAs.

**TABLE 1:** Genes associated with hypertelorism.

Genes	Description	Mouse line	Broad craniofacial phenotypic presentation	Hypertelorism in humans	References
Phenotype specific to the midfacial region					
*Alx4*	ALX homeobox 4	Homozygous spontaneous	Frontonasal dysplasia type 2; short nose, preaxial polydactyly, dorsal alopecia, bulging anterior cranium, open eyelids	Yes [PMID: 34098755, 32216639, 29681084]	PMID: 25673119
*Arsb*	Arylsulfatase B	*Arsb* ^*Y85H/Y85H*^	Coarse facial features, short stature	No (coarse face) [PMID: 21930407]	PMID: 36032399
		*Arsb* ^*−/−*^	Mucopolysaccharidosis type VI; broad head and short snout		PMID: 8710849
*Bmpr1a*	Bone morphogenetic protein receptor, type 1A	*P0-Cre;caBmpr1a* ^*F/F*^ (constitutively active)	Isolated sagittal synostosis, short snout	Yes, due to deletion of 10q22.3q23.2 (containing BMPR1A) [PMID: 20345475]	PMID: 22773757, 11536076
		*Mpz-Cre;dnBmpr1a* ^*F/F*^ (dominant negative)	Midline cleft (83%) or short snout and hypertelorism		PMID: 22773757
*Frg*	froggy	Homozygous spontaneous	Short snout, immature fusion of maxillary-premaxillary sutures	Not in humans	PMID: 26234751
*Gnas*	GNAS (guanine nucleotide binding protein, alpha stimulating) complex locus	*Wnt1-Cre;Gnas* ^*F/F*^	Domed cranium, absence of nasal capsule, cleft palate, abnormal maxilla and mandibular bones (missing condylar and coronoid processes, hypoplastic)	Yes [PMID: 15658617]	PMID: 26859889
*Idua*	Iduronidase, alpha-L	*Idua* ^*−/−*^	Mucopolysaccharidosis type I; broad face, short snout, dislocated hip, dysostosis	No (coarse face and low nasal bridge)	PMID: 9097952
*Lpar1*	Lysophosphatidic acid receptor 1	*Lpar1* ^*−/−*^	Short stature, osteoporosis	No	PMID: 21569876
		*Osx-Cre;Lpar1* ^*F/F*^	Osteoporosis, thinner cortical bone, short femur; craniofacial phenotype not investigated	-	PMID: 32330664
*Pfas*	Phosphoribosylformylglycinamidine synthase (FGAR amidotransferase)	Heterozygous spontaneous (*Pfas* ^*sofa/+*^)	Upturned short snout, premature closure of cranial synchondrosis, micro/anophthalmia	No	PMID: 26234751
*Tbx15*	T-box 15	*Tbx15* ^*de(H)/de(H);*^ *C57BL/6J* ^*ae/ae*^	Broad nasal area	Yes [PMID: 19068278]	PMID: 15652702
		*Tbx15* ^*LacZ/LacZ*^ (*Tbx15* ^*−/−*^)	Broad snout, short hypoplastic long bones		PMID: 15652702
*Tet1*	Tet methylcytosine dioxygenase 1	*Tet1* ^*−/−*^	Either midline cleft, bifid nose, or hypertelorism due to intracranial lipoma	No	PMID: 26989192, 22246904
*Tfap2a*	Transcription factor AP-2, alpha	*Tfap2a-Cre;Tfap2a* ^*F/F*^	Short concave nasal bone and snout, lack of interdigitation at the frontonasal suture, malocclusion	No [PMID: 19685247]	PMID: 14975718
		(*Wnt1-Cre;Tfap2a* ^*F/F*^does not have hypertelorism)			
*Tmem53*	Transmembrane protein 53	*Tmem53* ^*1bin/1bin*^(1 bp T insertion)	Hyperossification of calvaria, craniofacial hyperostosis, short limbs	Yes [PMID: 33824347]	PMID: 33824347
Phenotype broadly in the craniofacial region					
*B3glct*	Beta-3-glucosyltransferase	*B3glct* ^*D11-12/D11-12*^	Peters plus syndrome; short snout, domed cranium, deviated nose (nasal asymmetry), soft tissue syndactyly	Yes [PMID: 23161355, 23889335]	PMID: 31600785
*Bey*	Bulgy-eye	Bey/+ (altered regulation of *Fgf3* and *Fgf4* expression)	Crouzon syndrome; pansynostosis, domed cranium, short snout and maxilla	Synthetic phenotypic maker (not in humans)	PMID: 9626498
*Chd8*	Chromodomain helicase DNA binding protein 8	*Chd8* ^*+/−*^	Increased brain size	Yes [PMID: 24998929]	PMID: 29668850
*Crkl*	v-crk avian sarcoma virus CT10 oncogene homolog-like	*Crkl* ^*snoopy/snoopy*^ (T>C at Chr 16: 17452790)	Agnathia or hypognathia, maxillary hypoplasia, holoprosencephaly, microphthalmia or anophthalmia	Yes, due to deletion of 22q11.2 (containing *TBX11*, *CRKL1*, and *MAPK1*) [PMID: 27629806, 27182748]	PMID: 25565927
		*Crkl* ^*−/−*^	22q11.2 deletion syndrome (DiGeorge syndrome); shorter/wider head		PMID: 11242111
*Dnmt3b*	DNA methyltransferase 3B	Point mutation	Immunodeficiency, centromeric region instability, facial anomalies (ICF) syndrome; short snout	Yes [PMID: 21120685, 17893117]	PMID: 16501171
*Fgfr1*	Fibroblast growth factor receptor 1	*Fgfr1* ^*P252R/+*^	Pfeifer syndrome; craniosynostosis	Yes [PMID: 32510873]	PMID: 10942429
*Fgfr2*	Fibroblast growth factor receptor 2	*Fgfr2* ^*C342Y/+*^ (gain-of function)	Crouzon syndrome; craniosynostosis (coronal, lambdoid)	No hypertelorism with p.C342Y variants [PMID: 28901406]	PMID: 15459175, 15316116
				Apert syndrome with p.P253R and p.S252W exhibit hypertelorism [PMID: 25867380]	
*Fgfr3*	Fibroblast growth factor receptor 3	*Fgfr3* ^*P244R/P244R*^	Muenke syndrome; domed cranium, short and skewed snout, maxillary retrognathia, class III malocclusion, delayed ossification; no coronal synostosis	Yes [PMID: 32510873, 2133754]	PMID: 19086028
*Kif3a*	Kinesin family member 3A	*Wnt1Cre/+; Kif3a* ^*fl/fl*^	Hydrocephalus, isolated craniosynostosis, hypertelorism, and clefting	No	PMID: 24887031
*Kras*	Kirsten rat sarcoma viral oncogene homolog	*Kras* ^*V14I/V14I*^	Noonan syndrome; short stature, short snout, wider/higher/shorter cranium	Yes [PMID: 32021610]	PMID: 25359213
*Lrp2*	Low density lipoprotein receptor-related protein 2	*Lrp2* ^*−/−*^	Donnai-Barrow syndrome Midline cleft, micro/anophthalmia, holoprosencephaly	Yes [PMID: 36777721, 34872573]	PMID: 34463328
*Mks1*	MKS transition zone complex subunit 1	*Mks1* ^*LacZ/LacZ*^ (*Mks1* ^*−/−*^)	Meckel syndrome; holoprosencephaly, micrognathia (7/35), cleft lip (4/35), micro/anophthalmia (10/35); hypertelorism only 1/35	No	PMID: 23454480
*Ptch1*	Patched 1	*Ptch1* ^*−/−*^	Gorlin-Goltz syndrome; lambdoid synostosis, domed cranium	Yes [PMID: 31533758, 2511723]	PMID: 23897749
		*Ptch1* ^*mes/mes*^	Short snout (short head)		PMID: 8830098
*Ptpn11* (a.k.a. *Shp2*)	Protein tyrosine phosphatase, non-receptor type 11	*Ptpn11* ^*T468M/+*^	Noonan syndrome; short head	Yes [PMID: 32164556, 19864201]	PMID: 25288766, 21339643
		*Ptpn11* ^*Y279C/+*^	LEOPARD syndrome or Noonan syndrome; short stature, slanted eye, flattened nasal bridge	Yes [PMID: 18241070, 19768645]	PMID: 21339643
*Raf1*	v-raf-leukemia viral oncogene 1	*Raf1* ^*L613V/+*^	Noonan syndrome; cranium with decreased height and increased width	Yes [PMID: 20052757]	PMID: 21339642
*Rreb1*	ras responsive element binding protein 1	*Rreb1* ^*+/−*^	Short snout, Noonan-spectrum of syndromes	Yes [PMID: 32938917]	PMID: 32938917
*Ror2*	Receptor tyrosine kinase-like orphan receptor 2	*Ror2* ^*−/−*^	Robinow syndrome; midfacial hypoplasia, cleft palate, brachydactyly/polydactyly, short limbs	Yes [PMID: 35344616, 33496066]	PMID: 14745966
*Sh3pxd2b*	SH3 and PX domains 2B	*Sh3pxd2b* ^*−/−*^	Frank-Ter Haar syndrome; opened sagittal suture at 5 months of age, short cranium, micrognathia, thoracic kyphosis, lumbosacral lordosis	Yes [PMID: 35205281]	PMID: 20137777
*Sos1*	SOS Ras/Rac guanine nucleotide exchange factor 1	*Sos1* ^*E846K/+*^ and *Sos1* ^*E846K/E846K*^ (gain of function)	Noonan syndrome 4; blunt short snout	Yes [PMID: 27521173]	PMID: 21041952

Next, to identify common BP, CC, and MF in the genes, we conducted a GO analysis and found that the hypertelorism-related genes were significantly enriched in regulation of cell proliferation, neurogenesis, and various tissues development in BP; kinesin II complex, axonal growth cone, plasma membrane raft, caveola, dendritic shaft, ciliary transition zone, receptor complex, dendrite, dendritic tree, neuronal cell body, and Golgi apparatus in CC; and FGF-activated receptor activity, adenylate cyclase activator activity, cyclase regulator activity, D1 dopamine receptor binding, FGF binding, peptide hormone receptor binding, enzyme activator activity, transmembrane receptor protein kinase activity, growth factor binding, purine ribonucleotide triphosphate binding in MF (Figure 1D; Supplementary Table S4).

### 3.2 Prediction for miRNAs in the regulation of genes related to hypertelorism

To predict miRNAs regulating genes related to hypertelorism, we performed bioinformatic analyses with these 31 hypertelorism-related genes using the miRTarbase, miRbase, MirDB, and TargetScan, and found that miR-383-3p potentially regulated the expression of 19 hypertelorism-related genes (*Alx4*, *Arsb*, *B3glct*, *Chd8*, *Crkl*, *Fgfr1*, *Idua*, *Kif3a*, *Kras*, *Lpar1*, *Lrp2*, *Pfas*, *Ptch1*, *Ptpn11*, *Ror2*, *Rreb1*, *Sh3pxd2b*, *Tet1*, and *Tmem53*; *p* = 5.05 × 10^−5^); miR-6951-3p potentially regulated the expression of 18 hypertelorism-related genes (*Alx4*, *Bmpr1a*, *Chd8*, *Crkl*, *Dnmt3b*, *Fgfr1*, *Fgfr2*, *Gnas*, *Kif3a*, *Kras*, *Lpar1*, *Lrp2*, *Ptch1*, *Ror2*, *Rreb1*, *Sos1*, *Tet1*, and *Tfap2a*; *p* = 4.96 × 10^−4^); miR-7116-3p potentially regulated the expression of 18 hypertelorism-related genes (*Alx4*, *Bmpr1a*, *Chd8*, *Crkl*, *Dnmt3b*, *Fgfr1*, *Fgfr2*, *Gnas*, *Kif3a*, *Kras*, *Lpar1*, *Lrp2*, *Ptch1*, *Ror2*, *Rreb1*, *Sos1*, *Tet1*, and *Tfap2a*; *p* = 1.08 × 10^−6^); and miR-124-3p potentially regulated the expression of 17 hypertelorism-related genes (*Alx4*, *Bmpr1a*, *Chd8*, *Crkl*, *Fgfr1*, *Fgfr2*, *Gnas*, *Kif3a*, *Kras*, *Lpar1*, *Ptpn11*, *Raf1*, *Ror2*, *Rreb1*, *Sos1*, *Tbx15*, and *Tet1*; *p* = 2.23 × 10^−7^) (Figure 1E; Table 2).

**TABLE 2:** Candidate miRNAs associated with hypertelorism.

miRNA	*p* value	*q* value FDR bonferroni	Gene number	Hypertelorism-associated genes
miR-383-3p	5.05 × 10^−5^	2.92 × 10^−1^	19	*Alx4, Arsb, B3glct, Chd8, Crkl, Fgfr1, Idua, Kif3a, Kras, Lpar1, Lrp2, Pfas, Ptch1, Ptpn11, Ror2, Rreb1, Sh3pxd2b, Tet1, Tmem53*
miR-6951-3p	4.96 × 10^−4^	1.00	18	*Alx4, Bmpr1a, Chd8, Crkl, Dnmt3b, Fgfr1, Fgfr2, Gnas, Kif3a, Kras, Lpar1, Lrp2, Ptch1, Ror2, Rreb1, Sos1, Tet1, Tfap2a*
miR-7116-3p	1.08 × 10^−6^	6.22 × 10^−3^	18	*Alx4, Bmpr1a, Chd8, Crkl, Dnmt3b, Fgfr1, Fgfr2, Gnas, Kif3a, Kras, Lpar1, Lrp2, Ptch1, Ror2, Rreb1, Sos1, Tet1, Tfap2a*
miR-124-3p	2.23 × 10^−7^	1.29 × 10^−3^	17	*Alx4, Bmpr1a, Chd8, Crkl, Fgfr1, Fgfr2, Gnas, Kif3a, Kras, Lpar1, Ptpn11, Raf1, Ror2, Rreb1, Sos1, Tbx15, Tet1*
miR-669c-3p	1.26 × 10^−4^	7.32 × 10^−1^	10	*Chd8, Crkl, Kif3a, Lpar1, Pfas, Ptch1, Rreb1, Tbx15, Tet1, Tmem53*
miR-672-3p	1.20 × 10^−2^	1.00	5	*Crkl, Fgfr1, Kif3a, Ptch1, Tmem53*
miR-1231-5p	1.25 × 10^−3^	1.00	3	*Dnmt3b, Tfap2a, Tmem53*
miR-6905-5p	6.81 × 10^−4^	1.00	2	*Dnmt3b, Tfap2a*
miR-673-5p	3.22 × 10^−6^	1.86 × 10^−2^	9	*Sh3pxd2b, Chd8, Crkl, Kif3a, Kras, Lrp2, Ptch1, Ror2, Rreb1*
miR-7227-3p	2.85 × 10^−5^	1.65 × 10^−1^	8	*Alx4, Bmpr1a, Fgfr1, Kif3a, Kras, Rreb1, Tet1, Tmem53*
miR-875-3p	1.58 × 10^−5^	9.16 × 10^−2^	8	*Alx4, Bmpr1a, Fgfr1, Kif3a, Kras, Rreb1, Tet1, Tmem53*

### 3.3 Experimental validation for candidate miRNAs in cell proliferation and gene regulation

**FIGURE 2:**
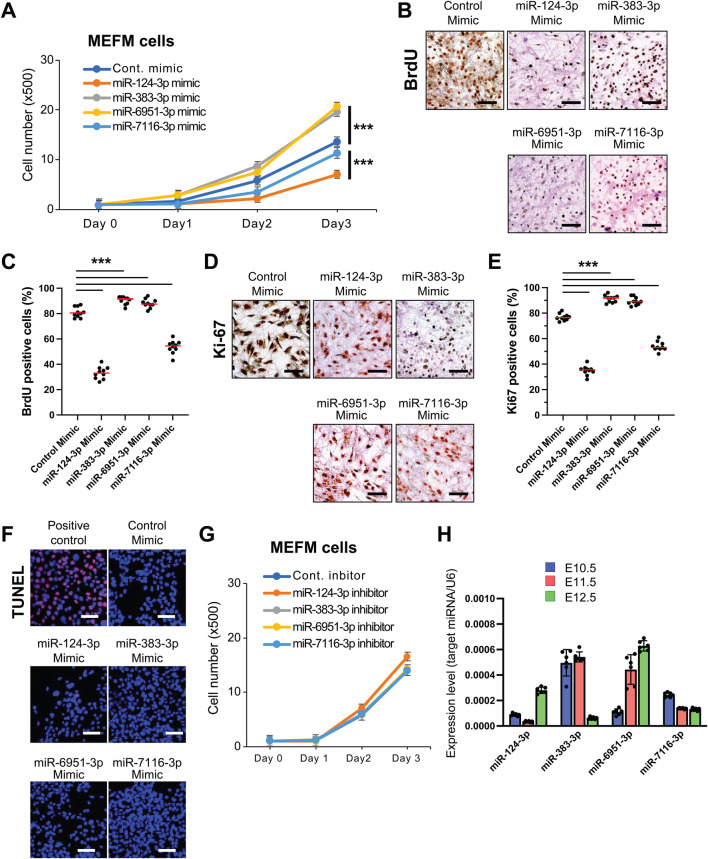
Effect of mimic and inhibitor of candidate miRNAs on cell proliferation and apoptosis in MEFM cells. **(A)** Cell proliferation assays in MEFM cells treated with the indicated miRNA mimic. ***p < 0.001. **(B)** BrdU incorporation assays in MEFM cells treated with the indicated miRNA mimic. Scale bars, 50 μm. **(C)** Quantification of the BrdU incorporation assays. ***p < 0.001. *n* = 10 field images from the cell culture dish per group. **(D)** Immunocytochemical analysis for Ki-67 in MEFM cells treated with the indicated miRNA mimic. Scale bars, 50 μm. **(E)** Quantification of the Ki-67 immunocytochemical analysis. ***p < 0.001. **p < 0.005. *n* = 10 field images from the cell culture dish per group. **(F)** TUNEL assays in MEFM cells treated with the indicated miRNA mimic or positive control. DAPI was used for nuclei staining. Scale bars, 50 μm. **(G)** Cell proliferation assays in MEFM cells treated with the indicated miRNA inhibitor. *p < 0.05. *n* = 6 per group. **(H)** Relative expression for the indicated miRNAs in the developing frontonasal region in C57BL/6J mice at E10.5 (light blue), E11.5 (pink), and E12.5 (light green). U6 (a housekeeping miRNA) was used for normalization. A total of six samples from different litters were analyzed.

We hypothesized that miRNAs related to hypertelorism increase cell proliferation by regulating the expression of target genes during frontonasal development. To investigate this, we examined the functional significance of candidate miRNAs in cell proliferation in primary cells isolated from the developing E10.5 mouse frontonasal prominence (MEFM cells) and O9-1 cells treated with specific mimics for each miRNA. Our findings showed that overexpression of miR-383-3p or miR-6951-3p significantly enhanced cell proliferation (Figures 2A–E; Supplementary Figures S1A–E). We confirmed that these miRNA mimics did not induce apoptosis in these cells (Figure 2F; Supplementary Figure S1F). Conversely, specific inhibitors for miR-383-3p or miR-6951-3p did not affect cell proliferation in these cells (Figure 2G; Supplementary Figure S1G). Notably, overexpression of either miR-124-3p or miR-7116-3p was inhibited cell proliferation in both MEFM and O9-1 cells (Figure 2; Supplementary Figure S1); therefore, we excluded miR-124-3p and miR-7116-3p from our list of candidate miRNAs associated with hypertelorism. These miRNAs were expressed at relatively low levels during frontonasal development in C57BL6/J mice at E10.5-E12.5 (Figure 2H).

**FIGURE 3:**
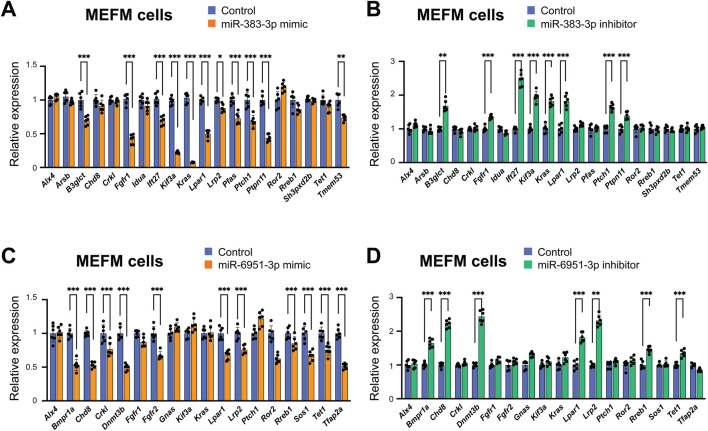
Gene regulation by candidate miRNAs in MEFM cells. **(A,B)** Quantitative RT-PCR for target hypertelorism-related genes in MEFM cells treated with miR-383-3p mimic **(A)** or inhibitor **(B)** for 24 h *p < 0.05, **p < 0.01. *n* = 6 per group. **(C,D)** Quantitative RT-PCR for target hypertelorism-related genes in MEFM cells treated with miR-6951-3p mimic **(C)** or inhibitor **(D)** for 24 h *p < 0.05, **p < 0.01. *n* = 6 per group.

Next, we validated the predicted miRNA-gene regulation for miR-383-3p and miR-6951-3p in MEFM and O9-1 cells after treatment with their respective mimics. The miR-383-3p mimic significantly downregulated the expression of *B3glct*, *Fgfr1*, *Ift27*, *Kif3a*, *Kras*, *Lpar1*, *Lrp2*, *Pfas*, *Ptch1*, *Ptpn11*, and *Tmem53*, while the miR-6951-3p mimic significantly downregulated the expression of *Bmpr1a*, *Chd8*, *Crkl*, *Dnmt3b*, *Fgfr2*, *Lpar1*, *Lrp2*, *Rreb1*, *Sos1*, *Tet1*, and *Tfap2a* in these cells (Figures 3A,C; Supplementary Figures S2A,C). In contrast, the miR-383-3p inhibitor significantly upregulated the expression of *B3glct*, *Fgfr1*, *Ift27*, *Kif3a*, *Kras*, *Lpar1*, *Ptch1*, and *Ptpn11*. Similarly, the miR-6951-3p inhibitor significantly upregulated the expression of *Bmpr1a*, *Chd8*, *Dnmt3b*, *Lpar1*, *Lrp2*, *Rreb1*, and *Tet1* in these cells (Figures 3B,D; Supplementary Figures S2B,D). Thus, miR-383-3p regulates the expression of *B3glct*, *Fgfr1*, *Ift27*, *Kif3a*, *Kras*, *Lpar1*, *Ptch1*, and *Ptpn11* in a dose-dependent manner, whereas miR-6951-3p regulates the expression of *Bmpr1a*, *Chd8*, *Dnmt3b*, *Lpar1*, *Lrp2*, *Rreb1*, and *Tet1*. Notably, *Lpar1* was an only gene regulated by both miR-383-3p and miR6951-3p.

## 4 Discussion

Craniofacial birth defects occur in a syndromic and/or nonsyndromic manner. Facial shape widely varies among individuals; therefore, an excessive difference from the mean or median is considered abnormal. Syndromic cases are caused mainly by monogenic and show a large effect size. On the other hand, nonsyndromic cases are caused by a complex interplay of genetic and epigenetic factors. These epigenetic factors, influenced by various conditions, including aging, nutrition, chemical/drug exposure, alcohol consumption, and smoking, contribute to developmental defects in humans and mice (Suzuki et al., 2016). Our bioinformatic analyses revealed that miR-383-3p and miR-6951-3p potentially regulate the expression of causative genes for hypertelorism in mice. Increasing evidence reveals miRNA functions under pathological conditions, but none are in FNP development. miR-383-3p is downregulated and induces cell growth in some types of cancer in humans (Jafarzadeh et al., 2022). It suppresses the epithelial-mesenchymal transition through the SHH-GLI family zinc finger 1 (GLI1) pathway in hepatocellular carcinoma (Chen et al., 2017) and through downregulation of the methylcrotonyl-CoA carboxylase subunit 1 (MCCC1) expression in malignant melanoma (Zhang et al., 2019) and gastric cancer (Huang et al., 2015). Interestingly, the expression of miR-383-3p is reduced in cardiomyocytes under a hypoxia/reoxygenation injury condition, leading to upregulation of the phosphatase and tensin homolog (PTEN)–phosphatidylinositol 3-kinase (PIK3) - AKT serine/threonine kinase signaling pathway, which increases apoptotic cell death (Zeng et al., 2022). By contrast, the function and expression of miR-6951-3p under physiological and pathological conditions are currently unknown. We found that cell proliferation was activated by overexpression of miR-383-3p and miR-6951-3p in MEFM and O9-1 cells. In contrast, although miR-124-3p and miR-7116-3p were predicted to be pathogenic miRNAs for hypertelorism, overexpression of miR-124-3p and miR-7116-3p inhibited cell proliferation in MEFM and O9-1 cells, suggesting that they are related to hypotelorism, an abnormally decreased distance between eyes. This observation is consistent with our previous findings in mouse lip mesenchymal cells (Suzuki et al., 2019), suggesting that induced miR-124-3p may lead to various craniofacial anomalies.

In this study, we curated genes associated with hypertelorism and predicted miRNAs that may regulate these genes. Our bioinformatic analyses identified miRNA-gene regulatory networks that could help uncover the causative mechanisms related to frontonasal anomalies. Our results show that several hypertelorism-related genes are uniquely and specifically regulated by miR-383-3p and miR-6951-3p. Among them, mice with a deficiency for *Fgfr1* (Zhou et al., 2000), *Fgfr2* (Eswarakumar et al., 2004), *Kif3a* (Liu et al., 2014), *Kras* (Hernandez-Porras et al., 2014), *Ptch1* (Sweet et al., 1996), *Rreb1* (Kent et al., 2020), *Sos1* (Chen et al., 2010), and *Tfap2a* (Nelson and Williams, 2004) show excessive proliferation during midfacial development, resulting in hypertelorism, suggesting that mimics of miR-383-3p and miR-6951-3p activate cell proliferation through suppression of these genes. Although it remains unknown why only the frontonasal region grows excessively under some conditions, our study suggests that some miRNAs, which are either specifically or broadly expressed, play a crucial role in FNP development and hypertelorism. Among the target genes, *Lpar1* was regulated by both miR-383-3p and miR-6951-3p. LPAR1 is widely expressed in multiple organs and embryonic tissues under physiological and pathological conditions (Boucharaba et al., 2004; Wu et al., 2019). Interestingly, mice deficient for *Lpar1*, one of the G-coupled lysophosphatidic acid (LPA) receptors, exhibit growth retardation, osteoporotic bones with low bone mass, calcification defects, and orbital hypertelorism (Gennero et al., 2011; Contos et al., 2000). LPA accelerates cell proliferation in cultured human skin-derived neural crest-like stem cells, and knockdown of *Lpar1* suppresses their cell proliferation and migration (Liu et al., 2021). These results suggest that the role of *Lrar1* and contribution to cell proliferation may depend on cell types. For example, it is known that LPA-LPAR signaling plays a role in bone development and osteogenic cell differentiation (Gennero et al., 2011; Alioli et al., 2020; David et al., 2014; Karagiosis and Karin, 2007). Taken together, increased cell proliferation may be caused by downregulation of multiple hypertelorism-related genes.

The KEGG analysis of hypertelorism-related genes revealed links to various cancers and carcinomas. Increased cell proliferation and migration are commonly observed in cancers characterized by rapid mass growth, and these processes are regulated by various growth factor signaling pathways, particularly FGF signaling (Turner and Grose, 2010). Our Gene Ontology (GO) results highlighted FGF signaling in this context. The pro-oncogene *Raf1* and its downstream RAS-MAPK pathways, including *Kras* and *Sos1*, were involved in all the pathways identified in the KEGG analysis. These genes are critical for regulating cell proliferation and growth in cancers. Although the circumstances surrounding cancer and embryonic development differ, there are shared pathways that may influence cell growth and migration in hypertelorism.

FGF signaling is also linked to carbon metabolism, which was emphasized in our KEGG analysis. FGF21, along with its receptor FGFR1c, and FGF1 with its receptors FGFRs, play roles in regulating glucose and lipid metabolism and energy expenditure (Markan and Potthoff, 2016; Angelin et al., 2012; Nies et al., 2022). Because glucose metabolism is crucial for ATP generation, an essential energy source for all cellular functions (Mulukutla et al., 2016), it is crucial for growth and proliferation. Interestingly, the regulation of glucose and lipid metabolism is influenced by miRNAs (Agbu and Carthew, 2021). For example, miR-130a, miR-130b, and miR-152 can suppress both glycolysis and mitochondrial metabolism (Bobbs et al., 2012). Notably, treatment with SU5402, an FGFR inhibitor, increases the expression of miR-130b during the gustation stage in the primitive streak of chick embryos (Bobbs et al., 2012). This suggests that FGF signaling inhibition may reduce glucose metabolism by altering miRNA expression. It is possible that several miRNAs associated with hypertelorism influence cellular metabolism and functions through various pathways.

Our findings hold promise for the development of new therapeutic and preventive approaches for craniofacial anomalies, motivating us to continue our research for a better understanding of these complex conditions. However, there are some limitations in this study. First, the functional significance of each miRNA evaluated in MEFM and O9-1 cells remains unclear *in vivo* regarding frontonasal anomalies. This study also focuses solely on increased mesenchymal cell proliferation as a potential mechanism for hypertelorism; therefore, future research should explore other potential mechanisms, such as orbital movement toward the midline, osteogenic differentiation, and influences from other structures. Moreover, the expression patterns and timing of miRNAs should be assessed *in vivo* using *in situ* hybridization once the appropriate probes are available.

## Data Availability

The original contributions presented in the study are included in the article/Supplementary Material, further inquiries can be directed to the corresponding author.
