# Supplementary material for: miR-383-3p and miR-6951-3p activate cell proliferation through the regulation of genes related to hypertelorism
Source: Front Cell Dev Biol. 2025 Jul 24;13:1587052. doi: 10.3389/fcell.2025.1587052 (PMC12333593; doi:10.3389/fcell.2025.1587052)
Supplement: Supplementary file 3 [file DataSheet1.pdf]

Figure S1

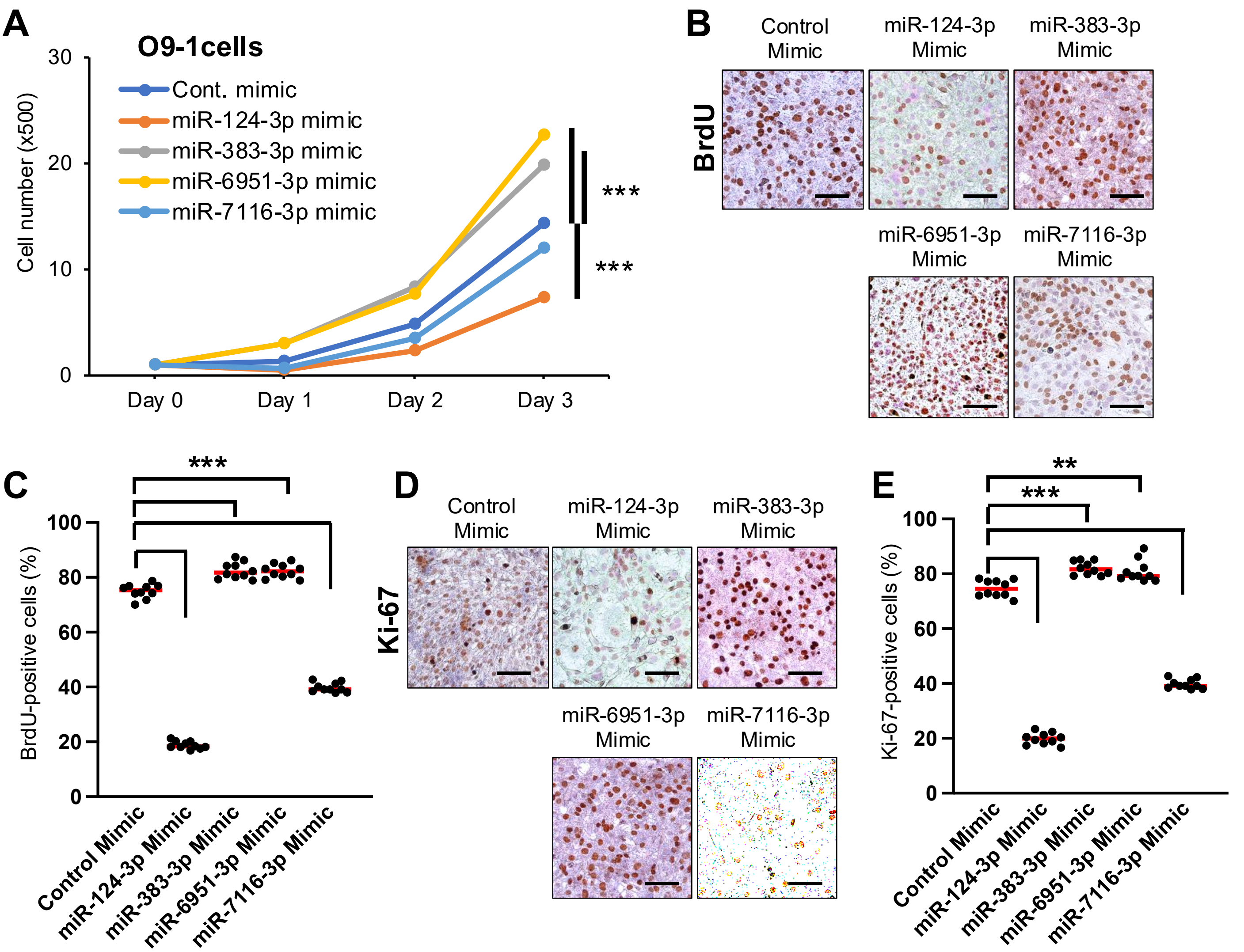

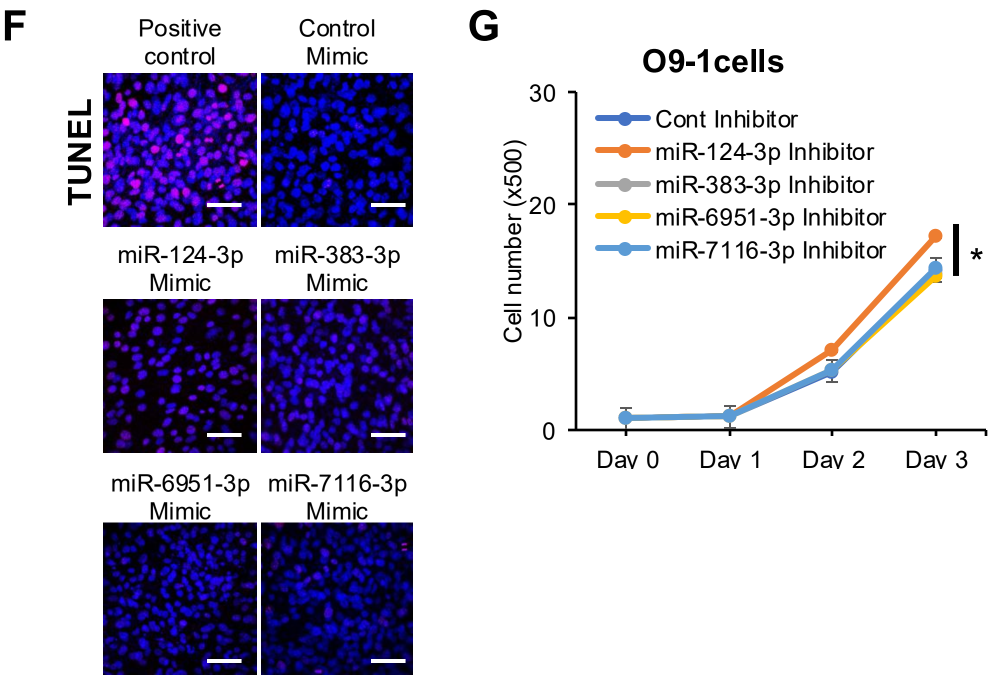

Figure S1. Effect of mimic and inhibitor of candidate miRNAs in O9-1 cells. (**A**) Cell proliferation assays in O9-1 cells treated with the indicated miRNA mimic. ***$p < 0.001$. (**B**) BrdU incorporation assays in O9-1 cells treated with the indicated miRNA mimic. Scale bars, 50 μm. (**C**) Quantification of the BrdU incorporation assays. ***$p < 0.001$. n=10 per group. (**D**) Immunocytochemical analysis for Ki-67 in O9-1 cells treated with the indicated miRNA mimic. Scale bars, 50 μm. (**E**) Quantification of the Ki-67 immunocytochemical analysis. ***$p < 0.001$. **$p < 0.005$. n=10 per group. (**F**) TUNEL assays in O9-1 cells treated with the indicated miRNA mimic or positive control. DAPI was used for nuclei staining. Scale bars, 50 μm. (**G**) Cell proliferation assays in O9-1 cells treated with the indicated miRNA inhibitor. *$p < 0.05$. n=6 per group.

Figure S2

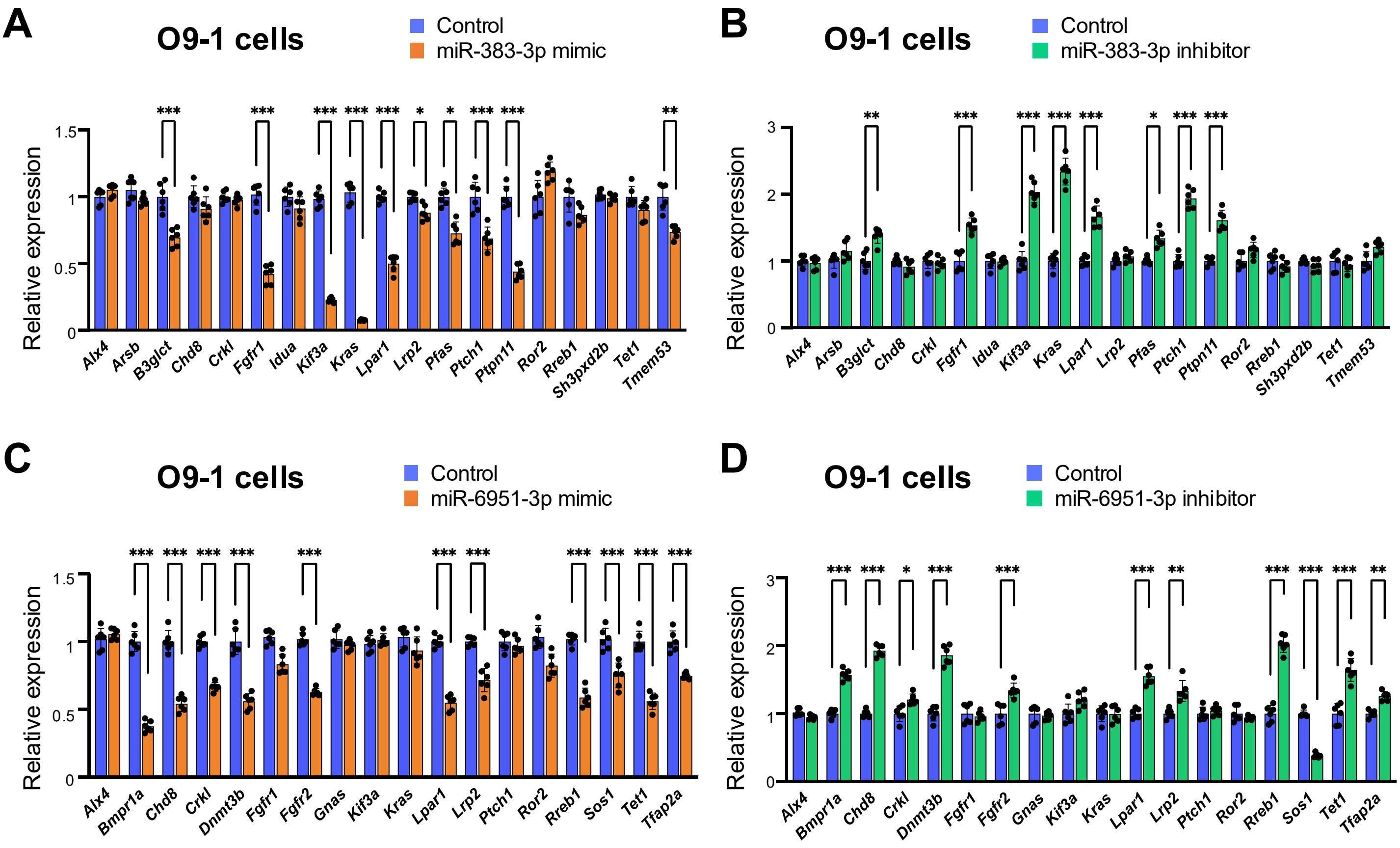

Figure S2. Gene regulation by candidate miRNAs in O9-1cells. (**A**, **B**) Quantitative RT-PCR for target hypertelorism-related genes in O9-1 cells treated with miR-383-3p mimic (A) or inhibitor (B) for 24 hours. **p* < 0.05, ***p* < 0.01. n=6 per group. (**C**, **D**) Quantitative RT-PCR for target hypertelorism-related genes in O9-1 cells treated with miR-6951-3p mimic (C) or inhibitor (D) for 24 hours. **p* < 0.05, ***p* < 0.01. n=6 per group.
